# Non-excitable fluorescent protein orthologs found in ctenophores

**DOI:** 10.1186/s12862-016-0738-5

**Published:** 2016-08-24

**Authors:** Warren R. Francis, Lynne M. Christianson, Meghan L. Powers, Christine E. Schnitzler, Steven H. D. Haddock

**Affiliations:** 1 Monterey Bay Aquarium Research Institute, 7700 Sandholdt Rd., 95039 Moss Landing, USA; 2 National Human Genome Research Institute, National Institutes of Health, 50 South Drive, Bethesda, 20892 USA; 3 Present address: Ludwig-Maximilians-Universität München, Munich, Germany; 4 Present address: Whitney Laboratory for Marine Bioscience, University of Florida, St. Augustine, Florida, 32080 USA

**Keywords:** Fluorescent protein, Ctenophore, Siphonophore, Transcriptome, Fluorescence, Haeckelia

## Abstract

**Background:**

Fluorescent proteins are optically active proteins found across many clades in metazoans. A fluorescent protein was recently identified in a ctenophore, but this has been suggested to derive from a cnidarian, raising again the question of origins of this group of proteins.

**Results:**

Through analysis of transcriptome data from 30 ctenophores, we identified a member of an orthologous group of proteins similar to fluorescent proteins in each of them, as well as in the genome of *Mnemiopsis leidyi*. These orthologs lack canonical residues involved in chromophore formation, suggesting another function.

**Conclusions:**

The phylogenetic position of the ctenophore protein family among fluorescent proteins suggests that this gene was present in the common ancestor of all ctenophores and that the fluorescent protein previously found in a ctenophore actually derives from a siphonophore.

**Electronic supplementary material:**

The online version of this article (doi:10.1186/s12862-016-0738-5) contains supplementary material, which is available to authorized users.

## Background

Fluorescent proteins (FPs) are abundant optical proteins in cnidarians [1–4] as well as other phyla, including arthropods [5–7] and chordates [8, 9]. They are structurally composed of a beta-barrel that surrounds the fluorophore [10]. The fluorophore is self-forming, requiring only molecular oxygen and the internal xYG residues of the protein. Because no other proteins or factors are required, FPs are used extensively in biotechnology for genetically encoded labels and reporters [11, 12].

In many cnidarians, such as the hydromedusa *Aequorea*, the fluorescent proteins are found as part of a binary system in conjunction with the bioluminescent photoproteins. In these natural resonant-transfer pairs, they modulate the color of the luminescence through energy transfer [1, 13–15]. On the other hand, many *non*-luminous cnidarians also exhibit a rainbow of fluorescence through diversification of the fluorescent protein set [4], potentially serving to attract prey [16].

It has long been known that most members of the phylum Ctenophora are bioluminescent [17–19] however it was not until more recently that a fluorescent protein was identified in the species *Haeckelia beehleri* [20]. The protein, which had the interesting property of photo-induced maturation, was clearly visible through the body, and the gene was reliably cloned from mRNA and expressed in bacteria. Because not all animal lineages had a sequenced member with an identified fluorescent protein, the position of this ctenophore protein in a phylogenetic tree had suggested it was very different from FPs of known cnidarians, particularly hydromedusa including *Aequorea victoria* [20]. However, in the published genome of *Mnemiopsis leidyi* [21], no fluorescent proteins were found, suggesting that ctenophores actually lack fluorescent protein genes [22].

Here we report evidence from the transcriptomes of 30 ctenophores that challenges both of these findings. Nearly every ctenophore examined expresses a fluorescent-protein-like (FPL) gene, including *Mnemiopsis leidyi* and *Pleurobrachia bachei*. These FPLs have substitutions in canonical residues involved in chromophore formation, suggesting that they either retained the ancestral function of the proto-FP, or may serve an entirely different function. Finally, by comparison to other FPs in the transcriptomes of more recently sequenced cnidarians, we determine that the FP from *Haeckelia beehleri* likely comes from a siphonophore through dietary uptake.

## Results

### Identification of a GFP-like protein in a ctenophore

We sequenced the transcriptomes of 28 ctenophores, and downloaded data for two other species. We had developed an automated protein search and identification strategy that searched for fluorescent proteins in hydromedusa transcriptomes, though this was automatically applied to the ctenophores as well. While most cnidarian fluorescent protein queries did not yield a BLAST hit in the ctenophore transcriptomes below the e-value threshold (10 ^−6^), we were surprised to find that one ctenophore (undescribed species *spT*) appeared to have a BLAST hit when Azami-Green FP was used as the query [23].

### Gene structure of the GFP-like protein

**Fig. 1:**
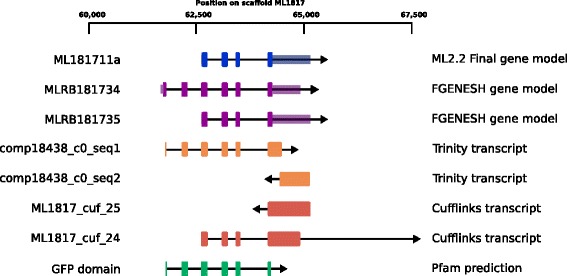
Schematic of the gene structure of *Mnemiopsis leidyi* FPL. Thick bars represent coding sequences while thin bars represent untranslated exon regions in the gene models. The final gene model in the ML2.2 release was taken from the FGENESH gene model MLRB181735, while instead MLRB181734 most accurately depicts the putative structure of the *M. leidyi* FPL gene. Trinity transcripts comp18438_c0_seq1 and comp18438_c0_seq2 overlap for 24 bp in the middle, despite going different directions. The cufflinks transcript ML1817_cuf_24 extends downstream for almost 20 kbp before finishing at an incorrectly positioned 28 bp exon. The unfiltered gene model MLRB181733 (not shown for clarity) extends for thousands of bases in both directions, bridging several other genes, and is likely to be an artifact

**Fig. 2:**
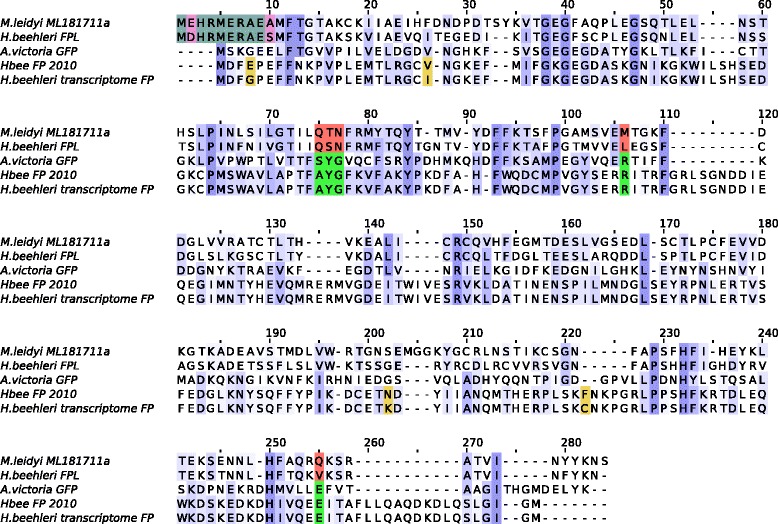
Multiple sequence alignment of FPLs. Multiple sequence alignment of selected fluorescent protein orthologs (FPLs) from ctenophores and fluorescent proteins. Sequences are as follows: *M. leidyi* ML181711a is the manually corrected protein from the *M. leidyi* genome; *H. beehleri* FPL is the ctenophore FPL from the *H. beehleri* transcriptome in this study; *A. victoria* GFP is the canonical GFP sequence; Hbee FP 2010 is the FP sequence described by Haddock et al. (2010); *H. beehleri* transcriptome FP was identified from the same *H. beehleri* transcriptome for this study. Intensity of blue color indicates conservation at the position. Residues involved in chromophore formation in FPs are shown in green, while the unconserved residues at the same positions in ctenophore FPLs are shown in red. The conserved N-terminal motif in the ctenophore FPLs is shown in gray, where unconserved residues are highlighted in pink. Amino acid differences between the two *H. beehleri* FPs are shown in yellow; there are only four differing positions, suggesting the two FPs are alleles

Based on this single ctenophore sequence, we re-examined the *Mnemiopsis leidyi* genome, which otherwise appeared to not have a fluorescent protein [22]. Using the undescribed species *spT* FPL as the query, we found an incomplete protein match (ML181711a) in the filtered gene models that had *Aequorea GFP* as the top BLAST hit. This protein appeared to have two exons missing at the N-terminus, although these exons were found in the unfiltered protein models (MLRB181734) (Fig. 1). Then, using the *M. leidyi* FPL as the query, orthologs in all other ctenophore species were easily found. In comparison to the transcriptome sequences, the *M. leidyi* protein model was missing a conserved N-terminal motif (Fig. 2). Another in-frame methionine that was 10 residues upstream was manually identified as the start codon to better correspond to the conserved N-terminal motif (approximately MxxRMERxxxxFxG). This motif was consistently found in all other ctenophore FPLs. Because some GFPs in the hydromedusa *Clytia hemispherica* are targeted to the mitochondria [24], we examined this FPL for targeting peptides. Although the ctenophore FPLs have a conserved N-terminal motif that alternates between charged and non-polar residues, neither SignalP nor TPpred2 predicted secretory or mitochondrial targeting based on this motif [25, 26].

### Ctenophore GFP-like proteins lack normal FP features

When the *M. leidyi* FPL was aligned to *A. victoria* FP, the most striking difference between the ctenophore FPL and cnidarian FP was the absence of the canonical xYG chromophore motif (Fig. 2, Additional files 1 and 2). In *M. leidyi*, this is replaced by QTN; it should be noted that the glutamine is completely conserved at this position in all other ctenophore species, and none of the other ctenophore sequences have the canonical chromophore residues. Furthermore, two critical residues involved in chromophore maturation, R96 and E222 (positions for *A. victoria*), are both substituted in all ctenophore FPLs. Based on known chromophore-formation mechanisms [27], we consider it highly unlikely that these proteins could form a fluorescent chromophore.

### Origin of the *Haeckelia beehleri* FP

**Fig. 3:**
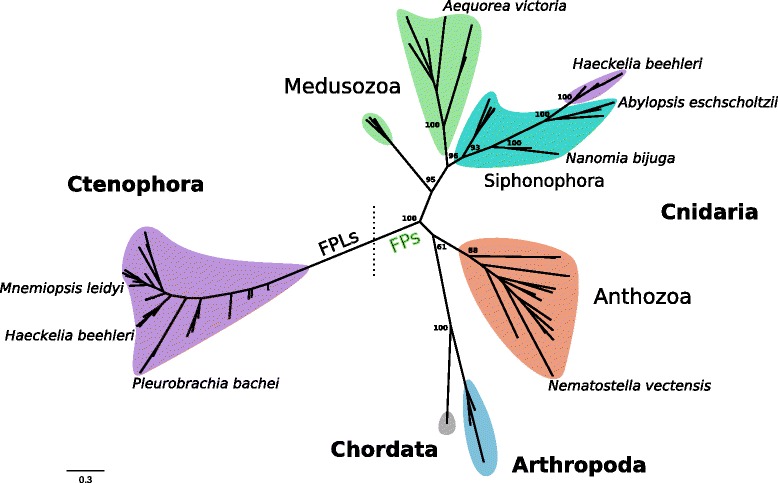
Phylogenetic tree of the FPLs. Phylogenetic tree of the FPLs with known fluorescent proteins from many clades. FPLs have only been found in ctenophores and are separated by the dotted line from true FPs in eumetazoans. Selected bootstrap values are shown. In all analyses ctenophore FPLs emerged as a monophyletic group with 100 bootstrap support, though support for many internal nodes within ctenophores was weak. The *H. beehleri* transcriptome contained transcripts which were placed in two locations on the tree: one FPL within Ctenophora, and one monophyletic group of proteins within Siphonophora that included the 2010 sequence as well as several transcripts from the transcriptome. Selected other species names are shown for reference

Further examination of the tree from the original *H. beehleri* FP publication [20] shows the ctenophore branch to be placed inside of the cnidarian clade, which is surprising given that ctenophores should be a separate clade. We carefully searched the transcriptome of *H. beehleri* and found two proteins, one more similar to the *M. leidyi* FPL and one matching (98 % identity) the FP identified previously by Haddock et al. [19]. To examine the molecular evolution of these proteins, we generated a phylogenetic tree of all of the FPLs from this study with known FPs (Fig. 3, Additional file 3). We were surprised to find a remarkable difference between the the positions of the previously cloned *H. beehleri* FP and the FPLs; the ctenophore FPLs formed a monophyletic group with 100 % bootstrap support at the base, while the previously cloned *H. beehleri* FP was included within the branch of siphonophores [28], a group of colonial cnidarians. This suggests that the FP in *H. beehleri* derives from a siphonophore, most likely as residual contamination from ingested material. Nonetheless, the same FP was found in the transcriptome of samples collected years apart, suggesting that this process is ongoing and may demonstrate some other biological reason that the mRNA of the FPs, as well as the proteins themselves, are apparently not degraded.

## Discussion

### Function of the FPLs

We consider it unlikely that these proteins are fluorescent for several reasons. Although nearly all ctenophores are bioluminescent, most are not fluorescent. Thus, even though the gene is being expressed, that is, found from transcriptomes, the fact that most species are not fluorescent suggests that these proteins are not contributing to any fluorescent phenotype. Additionally, important amino acids known to be involved in chromophore formation are absent in the ctenophore FPLs. While this does not strictly exclude the possibility that a chromophore could form in the native proteins, this is improbable.

The evolutionary origin of the GFP beta-barrel fold had been in question until a fragment of mouse nidogen was shown to have a remarkably similar structure [29]. Nidogen is a component of the extracellular matrix and is found in eumetazoans and the placozoan *T. adherens*, but no clear ortholog has been found in sponges or ctenophores. The G2 domain is involved in binding to perlecan and collagen, has the same secondary structure arrangement as GFP, and makes the same beta-barrel structure [29]. However, it was found that nidogens and FPs do not align at the sequence level, showing that sequences can differ dramatically while generating similar tertiary structures [29].

Because of the sequence divergence between the G2 domain and FPs, there is the remaining question of what would be required to generate a FP from a non-fluorescent precursor. Some unpublished attempts to engineer a fluorescent version of nidogen G2F were unable to generate a chromophore (Huiwang Ai and Robert Campbell, pers. comm.); their experiments included mutating the chromophore and maturation residues to those from GFP, swapping the alpha-helix of GFP into nidogen, and computational and random mutagenesis [30]. Their work suggests that many mutations are required to generate a chromophore and that the G2 domain of nidogen may very well have been optimized for orthogonal functions, thus making it unable to become a fluorescent protein. However, the divergence of the G2 domain from the pre-FP may have occurred long before the most recent common ancestor (MRCA) of eumetazoans (bilateral animals and cnidarians) and ctenophores, which may also explain the dramatic sequence divergence between the two proteins. The ctenophore proteins, with higher percentage identity to FPs than nidogen G2 domains, may be more amenable to such engineering experiments.

### Evolution of FPs and nidogen

It was argued that fluorescent proteins evolved once, rather than the G2 domain of a proto-nidogen becoming fluorescent in two bilaterian lineages, arthropods (copepods) and chordates (lancelets, only *Branchiostoma*) [5, 8]. If FPs evolved one time, then either most bilaterian lineages have lost the fluorescent proteins or a few have acquired it horizontally from a cnidarian. The gene tree (Fig. 3) suggests the first scenario, as bilaterian FPs form a monophyletic cluster indicating a single origin. The presence of fluorescent proteins across all major cnidarian groups [31, 32] suggests that the last common ancestor of the cnidarian crown group already had a functional fluorescent protein, and indeed this would have been present in the MRCA of eumetazoans as well. The extreme paucity of FPs across bilaterians suggests that the benefits to these marine groups may not apply to other taxa. For instance, there may have been selection against fluorescent phenotypes across many bilaterian groups, or selection for highly pigmented surfaces for UV protection or camouflage that may have removed the need for fluorescence.

Because the phylogenetic tree (Fig. 3) was unrooted, the outgroup of the fluorescent proteins cannot be assumed purely from the sequences provided. Therefore, the tree is compatible with two hypotheses for the relationship of ctenophores to the rest of the tree: the “coelenterata” hypothesis, monophyly of cnidarians and ctenophores, or to two alternative hypotheses, the “ctenophore-sister” or “sponge-sister” hypotheses, where ctenophores or sponges are sister group to all other metazoans, respectively. For the “coelenterata” hypothesis, if it is still assumed that there was a single evolutionary event to create fluorescent proteins, then ctenophores must have kept the orthologous proteins but lost the fluorescent function. This scheme also would require both one addition of fluorescence for all eumetazoans and one loss at ctenophores, and still offers no explanation of how fluorescence evolved in the first place. Detailed morphological analysis [33] and some recent phylogenomic analyses of metazoan proteins [21, 34] or gene content [21] find no support for the “coelenterata” hypothesis, although another study recovers this grouping [36]. Alternatively, given that there are no known FPs or FPLs from sponges or placozoans, the two alternative hypotheses, “ctenophore-sister” and “sponge-sister”, are both topologically equivalent for our dataset. Although the order of the basal groups remains a matter of complex debate [21, 35, 36], in either of two “sister” hypotheses, the implication is that ctenophores retained the ancestral protein, which later became fluorescent in the branch leading to bilaterians and cnidarians.

It was suggested that the G2 domain (and ultimately the full nidogen protein with the modern domain structure) emerged by an ancient duplication before the MRCA of eumetazoans [5]. One copy of this ancient protein became incorporated into nidogen, while the other changed to become fluorescent proteins in a pre-eumetazoan. The presence of nidogen with the G2 domain in a number of cnidarian genomes, such as *Nematostella vectensis* [37] and *Acropora digitifera* [38], and in bilateria indicates that both nidogen and fluorescent proteins were present at the MRCA of eumetazoans. Additionally, complete nidogen is also found in the genome of the placozoan *Trichoplax adherens* [39], showing that the G2 domain was already incorporated into nidogen before the emergence of eumetazoans. However, given that the G2 domain is a single exon in the placozoan *Trichoplax adherens* and the protein domain boundaries in the human gene nidogen-1 correspond to the exon boundaries as well, it is possible that the domain could have “jumped out” to become an isolated gene rather than individual domains joining together to form a larger protein, as is expected of nidogen. Thus, it is possible that FPs and FPLs were formed by copying out a single domain from a multidomain protein, even if that multidomain protein has since been lost multiple times. Further genomic analyses of non-bilaterian species and single-celled eukaryotes may uncover an older origin of this protein superfamily.

## Conclusions

Here we have demonstrated that proteins from the FP family are found in transcriptomes of 30 ctenophores. These FPLs lack the normal features of canonical fluorescent proteins and are unlikely to form fluorescent chromophores. The ctenophore FPLs form a clade within a tree of FPs from cnidarians and bilaterians, and may represent the ancestral condition of this protein before it became fluorescent in eumetazoans.

Unexpectedly, we found siphonophore FP sequences in the transcriptome of *Haeckelia beehleri*. The siphonophore FP genes found in this study are nearly identical to those reported years before [20], likely from the same species. Better identification of the prey may help to reveal why the exogenous RNA and the proteins evade degradation in *H. beehleri*.

## Methods

### Specimens

Specimens were collected either by trawl net, during blue-water dives, or using remotely-operated-underwater vehicles (ROVs), as described previously [40]. Animals were collected in the region bounded by 36° 44’ N 122° 02’W to the northeast and 35° 21’N 124° 00’W to the southwest. Operations were conducted under permit SC-4029 issued to SHD Haddock by the California Department of Fish and Wildlife. Species used are unprotected and unregulated, and no vertebrates or octopus were used, so the International and NIH ethics guidelines are not invoked. All samples were frozen in liquid nitrogen immediately following collection. All specimens were sequenced at the University of Utah using the Illumina HiSeq2000 platform paired-end with 100 cycles.

### Transcriptome assembly and analysis

All computations were done on a computer with two 2.5 GHz quad-core processors and 96 GB RAM. For each sample, raw RNAseq reads were processed as previously published [41]. Briefly, read order was randomized. Low-quality reads, adapters, and repeats were removed. For efficiency, subsets of reads were used to assemble transcriptomes. Assembly was done with both Velvet/Oases (v1.2.09/0.2.08) [42, 43] and Trinity (r2012-10-05) [44], though in nearly all cases better assemblies were obtained with Trinity. Transcripts from both assemblers were combined and redundant sequences were removed using the “sequniq” program in the GenomeTools package [45]. All BLAST searches were done using the NCBI BLAST 2.2.28+ package [46]. Ctenophore sequences used in analysis can be found at NCBI GenBank, with accessions: KT964712-KT964739, or found in Additional file 2.

### Reference data

Gene models, scaffolds, and proteins for the *Mnemiopsis leidyi* genome [21] v2.2 were downloaded from the *Mnemiopsis* Genome Portal (http://research.nhgri.nih.gov/mnemiopsis/). Gene models and transcripts for *Pleurobrachia bachei* genome v1.1 [34] were downloaded from the the Moroz Lab (http://moroz.hpc.ufl.edu/). Because there was not a systematic correspondence of the transcripts to proteins in the draft genome of *Pleurobrachia bachei*, further nucleotide analyses were excluded. Transcriptomic raw reads for *Abylopsis tetragona*, *Nanomia bijuga*, and *Physalia physalis* were downloaded from the NCBI SRA bioproject PRJNA205486 with accessions: SRR871525, SRR871527, and SRR871528, respectively [47]. Reads were assembled as above for the ctenophore transcriptomes.

### Sequence alignments and phylogenetic tree generation

Alignments for proteins sequences were created using MAFFT v7.029b, with L-INS-i parameters for accurate alignments [48]. The final alignment for tree generation contained 78 sequences with 428 positions, of which 44 % are gaps or undetermined positions. The phylogenetic tree for the fluorescent proteins was generated using RAxML-HPC-PTHREADS v7.2.8 [49], using the PROTCATWAG model for proteins and 1000 bootstrap replicates with the “rapid bootstrap” (-f a) algorithm and a random seed of 1234. Alternative models were tested (PROTGAMMAWAG and PROTCATLG,) though this resulted in nearly identical topology of the best tree with negligible changes to bootstrap support. Because some of the siphonophore sequences contained multiple tandem domains, these domains were split and treated as separate proteins for alignment and tree building; in all cases FP domains formed monophyletic groups by species, suggesting that domain splitting had minimal impact on the overall tree topology. An alternate alignment was generated with only a single domain from each multidomain FP, though the resulting tree had no meaningful difference in topology and still maintained monophyly of ctenophore FPLs and bilaterian FPs with 100 % bootstrap support.

## Additional files

Additional file 1 Fasta format of the alignment of all FPs and FPLs, for any alignment viewer such as SeaView. (TXT 34.4 kb)

Additional file 2 Unaligned proteins from ctenophores used to generate Figure 3. (TXT 19.5 kb)

Additional file 3 Newick format tree used for Figure 3, containing all bootstraps. (TXT 4.90 kb)
